# Patient-Driven Innovation for Mobile Mental Health Technology: Case Report of Symptom Tracking in Schizophrenia

**DOI:** 10.2196/mental.7911

**Published:** 2017-07-06

**Authors:** John Torous, Spencer Roux

**Affiliations:** ^1^ Digital Psychiatry Program Department of Psychiatry and Division of Clinical Informatics Beth Israel Deaconess Medical Center, Harvard Medical School Boston, MA United States

**Keywords:** schizophrenia, mobile health technology, smartphone, mhealth, serious mental illness, apps

## Abstract

This patient perspective piece presents an important case at the intersection of mobile health technology, mental health, and innovation. The potential of digital technologies to advance mental health is well known, although the challenges are being increasingly recognized. Making mobile health work for mental health will require broad collaborations. We already know that those who experience mental illness are excited by the potential technology, with many actively engaged in research, fundraising, advocacy, and entrepreneurial ventures. But we don’t always hear their voice as often as others. There is a clear advantage for their voice to be heard: so we can all learn from their experiences at the direct intersection of mental health and technology innovation. The case is cowritten with an individual with schizophrenia, who openly shares his name and personal experience with mental health technology in order to educate and inspire others. This paper is the first in JMIR Mental Health’s patient perspective series, and we welcome future contributions from those with lived experience.

## Introduction

This patient perspective piece presents an important case at the intersection of mobile health technology, mental health, and innovation. The potential for digital technologies to advance mental health is well known [1], although the challenges are being increasingly recognized [2]. Making mobile health work for mental health will require broad collaborations. We already know that those who experience mental illness are excited by the potential technology, with many actively engaged in research, fundraising, advocacy, and entrepreneurial ventures. But we do not always hear their voices as often as others. There is a clear advantage for their voices to be heard: we can all learn from their experiences at the direct intersection of mental health and technology innovation. This case is co-written with an individual with schizophrenia, who openly shares his name and personal experience with mental health technology in order to educate and inspire others. This paper is the first in JMIR Mental Health’s patient perspective series. We welcome future contributions from those with lived experience.

## Brief Case

Spencer Roux is a 28-year-old man who was diagnosed with schizophrenia approximately four years ago in 2013. In 2016, he was doing well and working 40 hours per week at his full-time job. However, in the fall of that year he began to notice that symptoms of auditory hallucinations were becoming more frequent. His psychiatrist recommended a change in his antipsychotic medication; however, before agreeing, Spencer wanted to be able to quantify the effects of this new medication.

In order to understand what effect the medication changes would have on his auditory hallucinations, he began theorizing how to track his symptoms. The primary question he sought to answer was, would higher doses of medication directly correspond to decreased symptoms of schizophrenia, in particular, hallucinations? To answer his question, he needed data to measure the number of hallucinations experienced per day during the time he was changing his medication. Spencer explained his plan to his psychiatrist and began looking for an easy way to count the number of hallucinations he was having per day.

He first investigated whether smartphone apps could serve as a tally counter, where each time he had a hallucination he could press the screen and log the event. But he quickly found that it was not convenient to use an app because unlocking his smartphone, opening the app, and pushing the digital on-screen counter was cumbersome and often impractical in many social settings. Searching for a better solution, he opted to use an actual tally counter to track his symptoms directly. Because he wanted to be able to access and store his data easily, mechanical tally counters did not match his needs. Instead he researched and purchased a smart tally counter, a digital device that, with the push of a button, wirelessly transmitted the current count to an online portal where he could later access it. While there are numerous digital tally counters available, Spencer selected one after seeing it in use at a local retail establishment and testing it there himself. This digital tally counter was convenient to use as it was easy to access, did not require frequent battery charging, and offered easy access to automatically time-stamped data which could be analyzed later.

**Figure 1:**
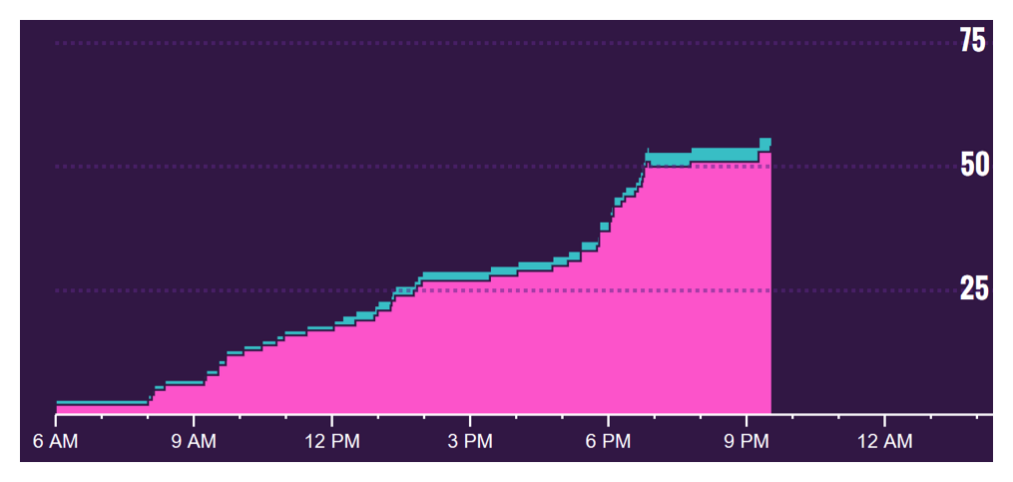
Cumulative sum and temporal distribution of auditory hallucinations (colored pink) on one particular day as recorded by button presses on the tally counter.

**Figure 2:**
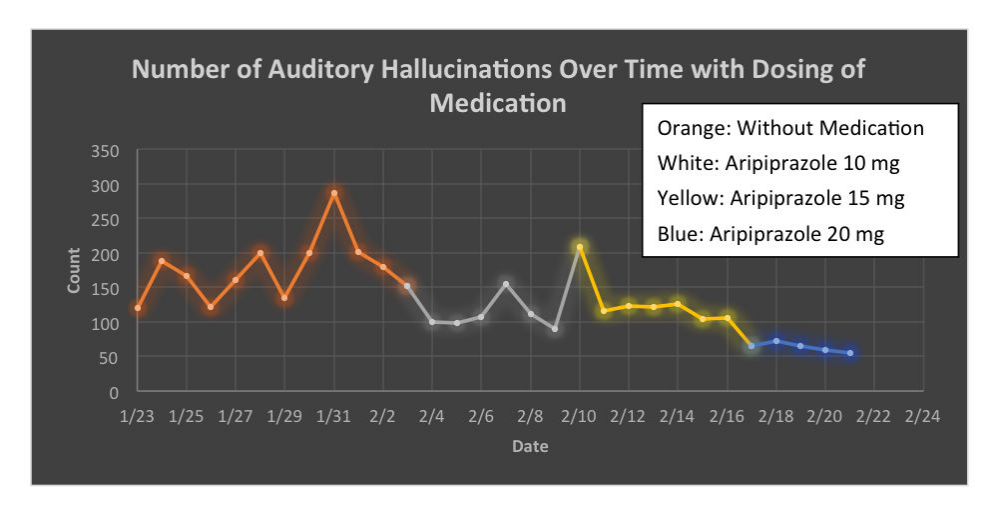
Daily frequency of auditory hallucinations as recorded by the tally counter across time and different doses of medications.

After buying this tally counter, he and his psychiatrist began the planned medication change. Over the course of the next four months, every time he had an auditory hallucination, Spencer simply recorded the instance with a press of a button with the digital counter. Below are the results for the first month. Spencer was able to access his own data whenever he wished and began to create graphs of his results, shown in Figures 1 and 2. These figures directly reflect Spencer’s efforts and were made in Excel (Microsoft Corp).

Spencer found the results to be exciting and informative. He shared them at each appointment with his psychiatrist and together they used the data to make shared and informed treatment decisions. As shown in Figure 2, Spencer noted that there was a correlation between higher doses of medication and fewer symptoms. Although he began this tally count monitoring with the hope that the lowest dose, or even no medication, would prove effective, the data seemed to tell a different story. While this was not the result Spencer was hoping for, having conducted his own experiment gave him confidence in the results and made him comfortable to remain on the dose that he and his psychiatrist agreed upon.

From the experience, Spencer learned it was possible not only to track and quantify his own mental health experience, but also that such data could be useful for assessing the effectiveness of treatment. The fact that the tally counter tracked the time of each button press allowed Spencer to gain insight into times of the day that were more triggering and to identify weekly fluctuations in symptoms.

## Brief Discussion

Spencer’s case is important in advancing recognition of innovation by those with lived experience of schizophrenia, challenging common notions of digital technology use for mental health and highlighting an important use case of mobile technology in mental health. While this is a single case so it is impossible to generalize to others, the lessons are useful for the entire field. Spencer’s case is notable for the combination of high engagement, strong interest in technology, and ability to accurately interpret symptoms and, thus, is not applicable to all patients. However, the broad themes of this case are of direct relevance to the entire field.

Creating technology solutions for those with the lived experience of schizophrenia must start with individuals like Spencer. While there has been much attention to the efforts of those with diabetes to use innovative wireless glucose tracking through the Nightscout project [3], there has been less attention to innovations for schizophrenia. When discussing the role of digital technologies for schizophrenia, many still question whether such technologies may worsen delusions or paranoia despite substantial evidence to the contrary [4,5]. Instead we need to be asking how we can learn from the experiences and innovations of those with schizophrenia to co-create digital solutions that are useful and impactful.

Spencer’s case also underscores how diverse digital solutions for mental health can be. There is increasing evidence that smartphone apps can be effective monitoring tools for mental health, in part because they are practical given that they are often within reach at all times [6]. But as Spencer’s case shows, sometimes a smartphone app is not the solution. There is also growing excitement about the potential of big data in mental health and using smartphones and sensors to gather tremendous amounts of data from those with mental illness, in order to uncover new insights [7]. Passive data, sensor data gathered without active user engagement (e.g., step count collected automatically by a fitness tracker versus active button presses on a tally counter), is an accelerating area of health research now. But as Spencer’s case shows, sometimes collecting the right data is more important than collecting a lot of data. Sometimes active data, in the form of hundreds of button presses, is the right answer over passive data. This is not to say that smartphone apps, big data, and passive data are not important for mental health but rather that, in Spencer’s case, there was a different technology solution that worked well. Given that passive data solutions require minimal engagement, they are likely to become an important tool for many, although active data solutions should not be overlooked.

Finally, this case serves as a good example of how technology can successfully integrate with clinical care and of how innovative ideas can be created. Spencer did not approach his journey by wondering how he could use a tally counter to improve his mental health. Rather he began with a focused, relevant, and actionable question of how changes in medication dosage were impacting his symptoms. Starting with a question and need, he explored how technology could provide him the necessary data. This question and need guided him to the right technology solution and fueled him with the motivation. Also of note, Spencer worked directly with his psychiatrist, and the technology was used to augment an existing treatment relationship with clinically actionable data and not to disrupt or challenge his care.

Spencer’s case represents the unique experiences and journey of a single individual with schizophrenia. What worked well for him may not work well for others as may be the case with many n of 1 and single-case experiments. But the message that those with schizophrenia are innovators in mobile health technology is applicable to all and one that we must increasingly recognize and learn from. Spencer’s solution reminds us that while big data, passive data, and smartphone apps are current areas of rapid growth for mental health technology, what really matters is what really works. Spencer built a solution that worked for him and created an impactful change in his mental health. Future iterations of Spencer’s solution may include a wearable device, although for now his current solution is working well. As we continue to learn more from the leadership and innovation of those with mental illness, the future for digital technology in mental health will be even brighter.
